# Network Analysis: A Novel Approach to Understand Suicidal Behaviour

**DOI:** 10.3390/ijerph14030219

**Published:** 2017-02-23

**Authors:** Derek de Beurs

**Affiliations:** Netherlands Institute for Health Services Research (NIVEL), Otterstraat 118-124, 3513 CR Utrecht, The Netherlands; derekdebeurs@gmail.com

**Keywords:** suicide, network analysis, symptoms, personalized treatment

## Abstract

Although suicide is a major public health issue worldwide, we understand little of the onset and development of suicidal behaviour. Suicidal behaviour is argued to be the end result of the complex interaction between psychological, social and biological factors. Epidemiological studies resulted in a range of risk factors for suicidal behaviour, but we do not yet understand how their interaction increases the risk for suicidal behaviour. A new approach called network analysis can help us better understand this process as it allows us to visualize and quantify the complex association between many different symptoms or risk factors. A network analysis of data containing information on suicidal patients can help us understand how risk factors interact and how their interaction is related to suicidal thoughts and behaviour. A network perspective has been successfully applied to the field of depression and psychosis, but not yet to the field of suicidology. In this theoretical article, I will introduce the concept of network analysis to the field of suicide prevention, and offer directions for future applications and studies.

## 1. Introduction

### 1.1. The Complexity of Suicidal Behaviour

**Figure 1:**
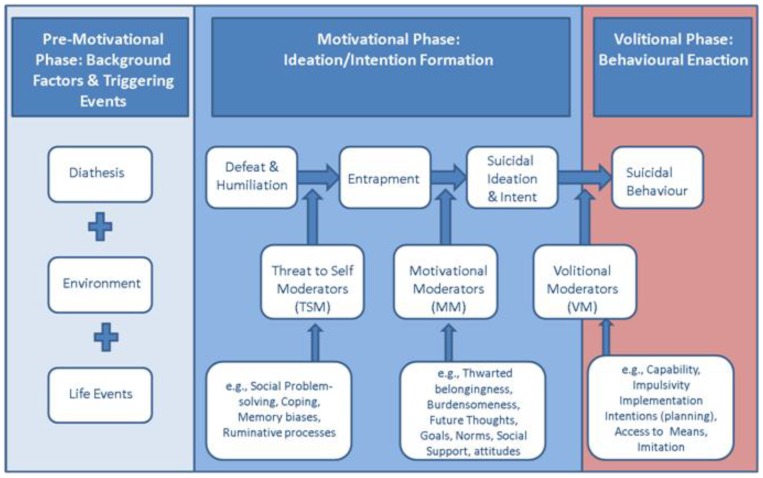
The Integrated Motivational Volitional (IMV) model of suicidal behaviour.

Suicide is a major public health issue worldwide [1]. It is the tenth leading cause of death, and in many countries, numbers have been increasing since the economic crisis in 2007 [2]. In the past years, many epidemiology studies have been done, resulting in several risk factors for suicidal behaviour such as gender and the presence of a psychiatric disorder [3]. Still, we understand little of how the interaction between these factors increases the risk for suicidal behaviour [4]. Traditional epidemiological analysis has resulted mostly in static, general risk factors such as age and gender. This is interesting from a public health care perspective, but not of much use for the individual patient, or his therapist. The notion that older males are at higher risk for suicide than younger females is not specific enough to be of clinical relevance. Recent models of suicidal behaviour agree that suicidal behaviour is the end result of the complex interaction between psychological, social and biological factors [4]. To help think about the complexity of suicidal behaviour, the Integrated Motivational Volitional Model (IMV) was developed (Figure 1) [4,5].

The IMV model conceptualizes suicide as a behaviour that develops through motivational and volitional phases. The motivational phase of the model describes the symptoms that are associated with the emergence of suicidal thoughts. Examples of motivational symptoms are entrapment, coping and rumination. By contrast, volitional phase symptoms are defined as those symptoms that govern the transition from suicidal thinking (ideation/intent) to suicidal behaviour, i.e., when a suicide attempt is more likely. Several studies have validated the central aspects of the model [4]. Entrapment and low levels of future thinking have been found to play a role in repeat suicidal behaviour [6]. Also, as predicted by the IMV model, volitional factors were found to differentiate between suicide attempters and people with only suicidal thoughts [7]. As these studies focused on separate elements of the IMV model, the next logical step is to test the correlations between the different factors simultaneously. To understand the complex interaction between the many variables as proposed in complex models such as the IMV model, a new psychometric technique called network analysis has been proposed.

### 1.2. A Network Approach to Psychopathology

A network approach to psychopathology states that symptoms such as fatigue and low mood interact with and cause each other [8]. It moves away from the traditional medical disease model, in which a disorder such as a Major Depressive Disorder (MDD) is the root cause of its observable symptoms (for example, low mood, fatigue or suicidal thoughts). This model holds in the field of somatic medicine, where a tumor can be the cause of observable symptoms such as headaches. As one can have a tumor without headaches and headaches without a tumor, one can separate the medical condition (a tumor) from the symptoms [8]. This perspective has not been empirically supported in the field of psychopathology. This may not be so strange, as it seems quite unlikely that one has a MDD without feeling blue [8]. The network approach to psychopathology offers an alternative explanation why psychiatric symptoms co-occur and has gained considerable recognition [9]. In the last years, statistical models and open-source software have been developed to estimate networks within the field of psychopathology. A recent review paper showed that the network approach has successfully been applied to the field of depression, psychosis and posttraumatic stress disorder (PTSD) [9]. Although suicidal thoughts or suicidal behaviour are not conceptualized as a mental disorder such as depression (although some authors have argued to do so [10]), I argue that the application of network analysis within the field of suicidology can help us better understand suicidal behaviour.

More specifically, I argue that a network perspective on suicidal behaviour can help us to (1) validate complex explanatory models of suicidal behaviour; (2) understand the differences between subgroups of suicidal patients; and (3) help the personalized treatment of suicidal behaviour

## 2. Application of Network Analysis within the Field of Suicide Prevention

### 2.1. Validating Complex Explanatory Models of Suicidal Behaviour

**Figure 2:**
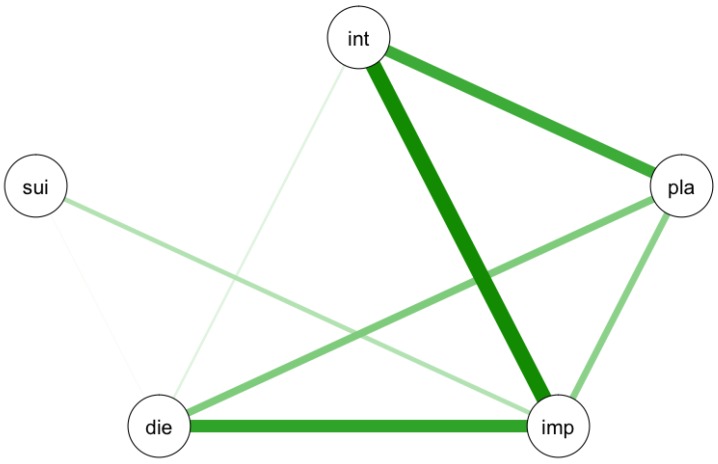
Hypothetical example of a network of suicidal symptoms. Int: intensity of suicidal thoughts; pla: concrete plans; imp: impulsivity; die: desire to die; sui: expectancy of suicidal behaviour. Green lines represent positive relationship. The thicker the line, the stronger the association.

As can be seen in Figure 1, the IMV model specifies the different components (symptoms) of the pre-motivational, motivational (ideation and intent formation) and volitional (behavioural enaction) phases of suicidality. According to the IMV model, suicidal ideation is likely to emerge as a result of the interaction between experiences of defeat and entrapment and specific moderators such as rumination or low levels of social support [5]. Via volitional moderators such as impulsivity or planning, a patient can move from the motivational to the volitional phase. The interaction between different elements of the model can be analyzed as a network. As an example, let us imagine we collected data within a sample of suicidal patients at one time point (i.e., the sample is cross-sectional). Patients had to state whether they disagreed or agreed with five statements on a five-point scale. The higher one scores, the more one agrees with the statement. The five statements included two motivational phase statements (I have a desire to die, I have intense suicidal thoughts) and three volitional phase statements (I act on impulse, I have concrete plans to commit suicide, I expect to show suicidal behaviour in the near future). The relation between the responses on the five statements can be estimated in a 5 × 5 correlation matrix. The freely available software package qgraph [11] allows us to visualize a correlation matrix as a network in which each node represents an item and each edge (line) a correlation. Within a network, two nodes are connected by a line if there is any relevant association between the two nodes. Whether there is a relevant association can be determined by using a partial correlation matrix. A partial correlation matrix is used rather than a correlation matrix to control for spurious associations and to derive at the conditional dependence structure. Two nodes that are not connected are independent when conditioning on other variables. For more details on the statistical background and methodology, I refer to other papers such as [11,12,13,14,15]. The output of a network visualization of the five items might look like Figure 2.

Figure 2 is not based on actual data, but merely illustrates that the relationship between different symptoms of the IMV model can be visualized as a network. In this hypothetical network, four symptoms (intensity of suicidal thoughts (int), concrete plans (pla), impulsivity (imp), desire to die (die)) are related to each other, although some more strongly than others. Next, only impulsivity is directly related to the expectancy of an attempt. More generally, one can see the network as the development of the suicidal process. One can calculate which symptoms are most central or important, i.e., connected to other symptoms [9]. These central symptoms are argued to be the most contagious, and change on that symptom is most likely to trigger a negative feedback loop. Early identification and early treatment can focus on this most contagious symptom, as any interventions will likely influence other symptoms, and this symptom may serve as a smoke detector for the start of a (new) suicidal crisis.

### 2.2. Understanding the Differences between Subgroups of Patients

**Figure 3:**
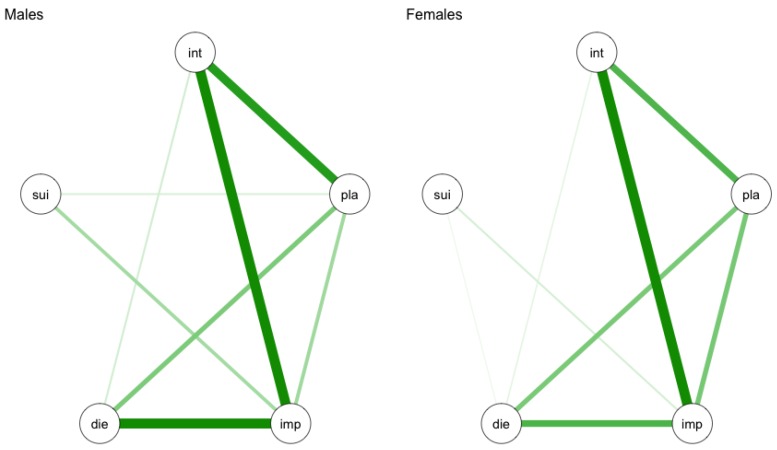
Hypothetical example of a network of suicidal symptoms for males and females. Int: intensity of suicidal thoughts; pla: concrete plans; imp: impulsivity; die: desire to die; sui: expectancy of suicidal behaviour. Green lines represent positive relationship. The thicker the line, the stronger the association.

Suicidal behaviour is likely to differ between subgroups of patients. For example, it is widely known that male suicidal behaviour differs from female suicidal behaviour [3,16]. An individual symptom analysis of longitudinal data revealed that stress resulted in elevated levels of suicidal ideation only within males [17]. Still, many studies do not take subgroup differences into account (e.g., [7,18,19]). This resulted in non-specific risk factors for suicidal behaviour which are of limited use when predicting suicidal behaviour. Understanding the difference in symptom structure between subgroups of patients will help in the development of more sensitive diagnostics. Network analysis allows for the comparison of the network structure of subgroups of patients. As an example, I simulate the same network as shown in Figure 3 separately for males and females.

In this example, one could argue that there are subtle differences in the network structure for males and females. Within the network of males, impulsivity seems to be more strongly related to the expectancy of an attempt when compared to females. Also within the network of males, suicidal behaviour and planning are directly related, whereas they do not seem to be related within the network of females. Additionally, it is possible to formally test the difference between networks using the R package Network Comparison Test (NCT) [20,21]. This non-empirical example gives insight into the potential of network analysis to better understand differences between subgroups of patients. These insights can then be translated into more calibrated diagnostic criteria to determine if somebody is at risk for suicidal behaviour.

### 2.3. Personalized Treatment Using Networks Based on Individual Data

When data is collected at multiple time points per patients (for example, via a mobile phone [22]) it is possible to form a unique network per patient. The network is then not based on group-level models, but on personal statistical models. Through this unique network, the patient can learn how different psychopathological symptoms interact within himself, and how to recognize the start of a new suicidal crisis. A patient can use his unique network as a tool to improve and personalize treatment. In a unique *n* = 1 study, a patient monitored her psychotic symptoms over the course of one year, answering 10 assessments a day, four days a week [23]. When presented as a network, the data provided clinically useful insights into the underlying symptom-to-symptom and symptom-to-context dynamics. It helped the patient to predict relapse, empowering the patient to gain more control over her recovery. Ideally, the unique network should be shared with professionals and significant others, and the three actors should consider the network structure when discussing therapy and safety planning. Such a research program on mobile phone data and suicidal behaviour has just been started at the Vrije Universiteit (VU) Amsterdam. The program is called CASPAR (Continuous Assessment for Suicide Prevention and Research) and will collect data among 30–60 patients receiving treatment of specialized health care. This data will be available at the end of 2017 and will offer the first opportunity to develop individual networks of suicide symptoms.

## 3. Discussion

Network analysis can help us better understand the complex interaction between symptoms that result in suicidal behaviour, and it can help us better differentiate between subgroups of patients. Network studies in other fields of psychiatry have resulted in new insights. For one, it was found that a more densely connected network at baseline predicts the presence of depression at follow-up [20]. Within the field of psychosis, network analysis revealed the relationship between childhood trauma and psychotic symptoms [24]. For individual suicidal patients, the main asset lies in the fact that they can gain insights into their own unique personalized network, making personalized treatment and safety planning much more likely. As suicidal behaviour is both trans-diagnostic and highly complex, a network analysis has much to offer to suicidologists, clinicians and suicidal patients.

### 3.1. Social Networks and Suicidal Behaviour

Within the field of sociology, a network theory of suicide was introduced as early as 1989 [25]. The authors re-evaluated Durkheim’s influential theory regarding the protective power of religion with regard to suicidal behaviour, and showed that the impact of religion is dependent on the strength of the social network it offers. Within their framework, a network theory of suicide does not exist in the relation between individual symptoms, but rather in terms of social capital and suicidal behaviour [26]. As the work of Durkheim and contemporary sociologists is highly influential in the field of suicidology, I want to emphasize that this network theory is definitely relevant, with a large tradition of research behind it [26]. It does, however, differ largely from the psychometric network analysis discussed in this paper.

### 3.2. Future Studies

For the application of network analysis on suicidal behaviour, two things are needed: a large enough dataset containing relevant suicidal symptoms, and the software program R (R Foundation, Vienna, Austria). R has a steep learning curve, but R studio offers an intuitive user interface. Additionally, there have been many tutorial papers published, and the R package qgraph offers easy-to-use syntax [11]. Sites such as psychosystems.org offer a lively community of scientists using network analysis within the field of psychiatry.

Together with an international consortium of suicide researchers, I am reanalyzing national and international datasets with information on suicidal behaviour from a network perspective. This project will be called the SUPER project (Suicide Prevention by Extending Research). In the Netherlands, there are several large databases such as the NESDA (Netherlands Study of Depression and Anxiety [27]). The NESDA is a longitudinal cohort study that collected the depressive, anxiety and suicidal symptoms of about 3000 patients. Within the Scottish Wellbeing Study from the Suicidal Behaviour Research Laboratory, data was collected among 3500 Scottish adolescents on many different suicidal symptoms such as entrapment, defeat, social exclusion, intrusion of images, perceived burdensomeness, etc. Other datasets of interest are the National Inquiry into Suicide and Homicide of the University of Manchester, and the Belgium Self-Harm Database which contains data on over 15,000 patients treated for a suicide attempt in Belgian hospitals during a 26-year study period [28]. By re-analyzing these large datasets, I expect to better understand the interaction between many different suicidal symptoms, learn about the differences between male and female suicidal behaviour, and zoom in on the differences between depressed patients that show suicidal behaviour and depressed patients that do not.

As network analysis is a relatively new technique, there are some important disclaimers to make. For one, it is of importance to have large datasets before one can estimate a stable analysis. Although the psychometrics are still being tested, as a rule of thumb it is argued that you need at least as many observations as you have parameters. So, for 10 symptoms, you need at least 55 observations (10 nodes + 10 × 9/2 possible interactions), for 20 you need 210 and for 50 you need 1250 [15]. This means that when testing a complex model such as the IMV, one needs a large sample size. The only databases that are likely to be large enough are general population databases such as the Scottish Wellbeing Study. Within these kinds of data, one has to be aware of overly low levels of psychopathology within the sample. Items with a low level of variability (due to the low frequency of actual “sick” people in the database) will have a low centrality within the total sample. Comparing the network structure of the total sample with the network of people with higher scores on psychopathology items is then recommended [29]. Additionally, when estimating networks using on cross-sectional data, there is no direct evidence of causality [9]. Longitudinal studies have to prove that intervening on central symptoms indeed results in less psychopathology at follow-up.

I want to point to a recent article that combined network analysis with latent trait analysis [14]. It showed that latent analysis such as structural equation modeling still is very useful, and can add novel information when combined with network analysis. Also, many other statistical innovations are being developed. For example, a recent application of a machine learning algorithm using existing clinical data found indicators of patients who are likely to respond to specific antidepressants [30]. Finally, both the network theory and software are still being improved. I therefore recommend anyone interested in these kinds of analysis to closely follow the papers of the psychosystems.org group.

## 4. Conclusions

Network analysis can help us better understand suicidal behaviour as it allows to visualize and quantify the complex associations between many different symptoms and their relation with suicidal behaviour.
